# Factors affecting the implementation of complex and evolving technologies: multiple case study of intensity-modulated radiation therapy (IMRT) in Ontario, Canada

**DOI:** 10.1186/1472-6963-11-178

**Published:** 2011-07-31

**Authors:** Kate Bak, Mark J Dobrow, David Hodgson, Anthony Whitton

**Affiliations:** 1 Cancer Services & Policy Research Unit, Cancer Care Ontario, 620 University Avenue, Toronto, M5G 2L7, Canada; 2 Department of Radiation Oncology, Princess Margaret Hospital, 610 University Avenue, Toronto, M5G 2M9, Canada; 3 Radiation Treatment Program, Cancer Care Ontario, 620 University Avenue, Toronto, M5G 2L7, Canada; 4 Department of Health Policy, Management and Evaluation, University of Toronto, 155 College Street, Toronto, M5T 3M6, Canada

## Abstract

**Background:**

Research regarding the decision to adopt and implement technological innovations in radiation oncology is lacking. This is particularly problematic since these technologies are often complex and rapidly evolving, requiring ongoing revisiting of decisions regarding which technologies are the most appropriate to support. Variations in adoption and implementation decisions for new radiation technologies across cancer centres can impact patients' access to appropriate and innovative forms of radiation therapy. This study examines the key steps in the process of adopting and implementing intensity modulated radiation therapy (IMRT) in publicly funded cancer centres and identifies facilitating or impeding factors.

**Methods:**

A multiple case study design, utilizing document analysis and key informant interviews was employed. Four cancer centres in Ontario, Canada were selected and interviews were conducted with radiation oncologists, medical physicists, radiation therapists, and senior administrative leaders.

**Results:**

Eighteen key informants were interviewed. Overall, three centres made fair to excellent progress in the implementation of IMRT, while one centre achieved only limited implementation as of 2009. Key factors that influenced the extent of IMRT implementation were categorized as: 1) leadership, 2) training, expertise and standardization, 3) collaboration, 4) resources, and 5) resistance to change.

**Conclusion:**

A framework for the adoption and implementation of complex and evolving technologies is presented. It identifies the key factors that should be addressed by decision-makers at specific stages of the adoption/implementation process.

## Background

There has been a rapid increase in the development of complex technologies intended to deliver radiation therapy more accurately to a tumour. In particular, intensity modulated radiation therapy (IMRT) is a method of delivering radiation to tumours while sparing surrounding healthy structures from the radiation dose [1]. Over the last decade, several (mostly non-randomized) studies have demonstrated that IMRT can provide greater tumour control, reduced treatment related morbidity, and increased quality of life in a number of cancers [2,3]. It should be highlighted that IMRT is not a single, static technology, but rather is understood to be a technological improvement in the radiation delivery technique [1]. In comparison to the introduction of a drug or even a medical device, such as a computed tomography scanner into clinical practice, the introduction of IMRT is extremely complex due to the need for 1) simultaneous implementation of supplementary technology, such as imaging modalities and computer software, 2) multifaceted quality assurance programs for all new software, and 3) various training and educational requirements for the multi-disciplinary radiation team, which consists of radiation oncologists, physicists and radiation therapists [1,3,4].

The rapid and continuing evolution in radiation technology results in the treatments becoming "increasingly automated, complex and critically software reliant" [5], creating challenging questions for decision-makers tasked with making adoption and implementation decisions. As IMRT can be provided in varying service delivery contexts, with considerable differences in terms of the number of specific disease sites treated annually, staff expertise, available equipment, type of cancer patients and the radiation doses delivered to patients [6-9], these adoption/implementation decisions are not straight forward and can lead to variable access to appropriate treatment.

Research regarding the factors that contribute to the adoption and implementation of radiation oncology innovations is lacking. Most published work describing the steps required to implement IMRT, are technical in nature and are geared towards physics personnel [10-14]. Furthermore, these studies do not directly consider the 'adopting system factors', which consist of the characteristics of the innovation, the adopters, the process and the organization, all of which impact on the implementation, and eventual success, of innovations [15]. Publicly funded cancer centres may face particular challenges regarding the adoption of new radiation technologies, since they often have a mandate to treat patients from a large catchment area and consequently may face challenges providing equitable access to these services while managing radiation wait times with constrained resources [16,17]. In fact, at the onset of this study (September 2007) only six out of twelve cancer centres in Ontario, Canada were offering treatment with IMRT, despite repeated calls for province wide implementation [4].

Considering the increasingly large investments made towards health care technologies, not only in the radiation oncology field but in other disciplines, demand for sound adoption and implementation decisions will be ongoing. Gaining a better understanding of the adoption/implementation process for complex and evolving technologies can lead to improvements in workflow, safety, efficiency and equitable access to appropriate care [18]. An in-depth examination of the process of adopting and implementing IMRT in Ontario was undertaken, with a focus on identifying the key barriers and enablers. This study provides insights for other jurisdictions that are attempting to improve and standardize adoption and implementation decisions for IMRT and/or other similar complex and evolving technologies.

## Methods

**Figure 1:**
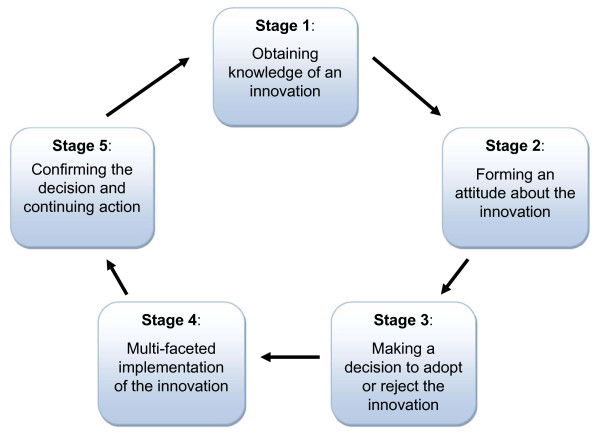
**Cyclical adoption/implementation cycle for complex and evolving technologies (Adapted from Rogers 1995)**.

A multiple case study design was employed because it is ideal for in-depth analysis of highly variable processes [19,20], such as the adoption and implementation of technological innovations like IMRT. Guided by Rogers' innovation adoption model [21] a conceptual framework (Figure 1) was developed for the implementation of complex and evolving technologies. Roger's model proposes five stages of adoption: 1) obtaining knowledge of an innovation, 2) formulating an attitude about the innovation, 3) making a decision to either adopt or reject the innovation, 4) implementing the innovation, 5) confirming the decision and continuing the action. While these stages are often depicted in a linear fashion, the conceptual framework presented in this study is cyclical and dynamic, as some researchers have proposed [22,23]. Purposive case selection criteria were used to select four cancer centres, which provided variation in geographical location, population served, size of the cancer centre, academic health sciences centre status, and treatment experience with IMRT (Table 1). A fifth cancer centre was used to pilot test an interview guide developed for the study.

**Table 1:** Case selection criteria

Case	IMRT Status	Academic Health Sciences Centre Status	Region	Population Covered*
Centre A	Established	Academic	Primarily urban	1 166 302

Centre B	Emergent	Non-academic	Primarily urban	1 885 587

Centre C	Emergent	Academic	Mixed urban-rural	480 853

Centre D	Established	Non-academic	Mixed urban-rural	559 056

*Estimates based on Local Health Integration Network (LHINs)

Two main methods were used to collect data including document analysis and key informant interviews. Document analysis was undertaken to gain a greater understanding of the cancer system context in Ontario, to provide contextual information for each of the cases, and to identify an initial set of factors potentially influencing the adoption/implementation decisions at each centre. Key informants were identified using both purposive and snowball sampling to ensure the inclusion of individuals who were actively involved in IMRT implementation. This included radiation oncologists, medical physicists, radiation therapists and senior cancer centre administrators. Semi-structured interviews were conducted in person, audio recorded and transcribed verbatim as text documents.

Coding of the interview transcripts was done using NVivo 7. Analytical categories in the coding structure were developed using a pragmatic approach [24] where themes were identified both deductively and inductively. The deductive component was related to the literature search, conceptual framework, interview guide and document analysis undertaken in the initial stages of the study. The deductively identified categories tend to be overarching themes that were applicable to general implementation studies (e.g., leadership, resources, etc), while analytical categories emerged inductively from transcript analysis using a constant comparison process where each item is systematically checked or compared to the remaining data [25]. A schematic process implementation map was developed for all four cancer centres as it can be "used to show precedence, parallel processes, and the passage of time" [26].

Definitions for anticipated themes were incorporated into the coding structure by the interviewer and reviewed by a second study member. The transcripts were then systematically coded by the interviewer; with a second study member independently coding a randomly selected sample (10% of the transcripts) at the outset and midpoint of the analysis. The coding results were compared and discussed to strengthen the coding strategy and enhance the consistency of data interpretation [24]. Data analysis was completed using a systematic process of reading, summarizing and categorizing the coded interview data into tabular format to explore various associations between themes. The emergent findings were further analyzed and discussed with study members. A small representative sample of quotations for key themes is presented below.

The interviews were audio-recorded and carried out by one interviewer to maintain internal consistency. The same individual transcribed all transcripts. Transcription accuracy was confirmed by listening to the audio recording while reading the transcripts. Ethics approval was obtained from the research ethics board at the University of Toronto and from all four cancer centres.

## Results

Case documents were collected from the key informants, the provincial cancer agency's Radiation Treatment Program and various websites. The majority of the documents discussed province-wide IMRT implementation rather than centre-specific initiatives. Seventeen of the 20 key informants invited to participate agreed to be interviewed. During the interview process one additional key informant was added through snowball sampling, increasing the accrued number of key informants to 18. Refer to Table 2 for additional key informant information. The interviews had an average duration of 45 minutes.

**Table 2:** Key informant participation by cancer centre and profession

Case	Profession
Centre A	1RO, 2MP, 1RT, 1SA

Centre B	1RO, 1MP, 1RT, 1SA

Centre C	1RO, 1MP, 1RT, 1SA

Centre D	1RO, 1MP, 1RT, 2SA

**Total:**	**4RO, 5MP, 4RT, 5SA**

RO = radiation oncologist, MP = medical physicist,

RT = radiation therapist, SA = senior administrator

### Case Contexts and Implementation Process

As of 2009, the four cancer centres were at various stages of progress in implementing IMRT (Table 3). Centres A, B and D had each made good but variable progress with IMRT implementation, while Centre C's progress was very limited over the same time period. Centre A was offering IMRT treatment to more than half of its patients requiring radiation and had expanded the IMRT program to a number of cancer sites (e.g., central nervous system, gastrointestinal, genitourinary, gynaecological, haematology, head and neck, lung, sarcoma, skin, and other cancer sites). Approximately 8% of patients with prostate cancer were being treated with IMRT at Centre B, while Centre D was offering the treatment to approximately 16% of patients with head and neck cancer and prostate cancer. At the time of this study Centre C had not yet started treating patients with IMRT.

**Table 3:** Implementation details for each of the cancer centres

	Implementation efforts begin	Started treating with IMRT	First disease site treated with IMRT	Subsequent disease sites treated	Percentage of patients treated with IMRT*
**Centre A**	Late 1990's	2001	Prostate	All indicated disease sites	56%

**Centre B**	2004	2008	Prostate	None	8%

**Centre C**	2004	2009	Head and Neck	None	0%

**Centre D**	2001	2004	Head and Neck	Prostate	16%

*(Quarter 4 of 2009/2010) Provincial average 22%

The main motivation for adopting/implementing IMRT at the four cancer centres was the recognition that IMRT was the new standard of care, which significantly improves patient outcomes. All four cancer centres set up implementation teams to help facilitate the process and held multidisciplinary workshops to introduce the implementation process and address concerns. The centres, to varying extents, relied on clinical trial protocols to guide the implementation. All four centres expressed the need for adequate physics support.

**Figure 2:**
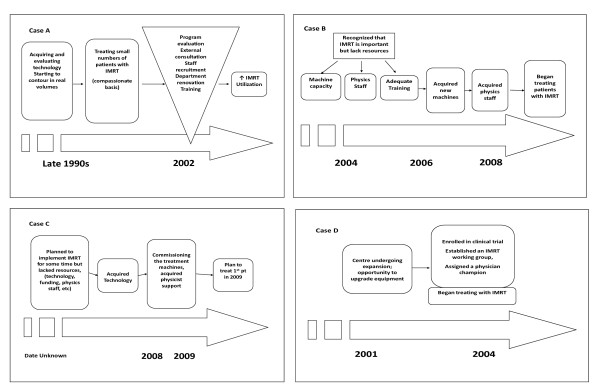
**Summary of the IMRT implementation process for each centre**. *The dates in these figures are estimates based on key informant's responses and do not correspond to specific boxes.

Centres A, B, and D took advantage of renovations or expansions occurring at their centres to implement IMRT, they sought advice from experts outside their centre prior to implementation, they had strong leadership and implementation champions and felt that implementing IMRT was important to meet the standard of care. While the implementation process at Centre C began around 2004 it was delayed due to a lack of resources (i.e., technology, time, funding), other competing priorities within the department and a lack of experienced physics staff. Additionally, the commissioning (the initial set-up testing by physicists) of the treatment planning took significantly longer than expected due to software glitches. Figure 2 documents the IMRT implementation process that occurred at each of the four centres.

### Implementation Factors

Considering the varying case contexts and progress at each of the four cancer centres over the period studied, a number of factors were identified that either enabled or impeded the adoption and implementation of IMRT. Each category of factors is described below.

#### Leadership

Leadership is defined as the ability of one individual to inspire, influence and challenge others to meet specified goals [27]. For the three cases where IMRT implementation progressed well, administrative leadership, including leaders outside of the radiation oncology department, played a prominent role. Where cancer centre leadership was a facilitator, the senior administrators interviewed were able to offer details and clearly describe their role in the IMRT adoption/implementation process at their respective centres. Where progress was slower, senior administrators were less aware of the centre's process or extent of implementation, suggesting a disconnect between the senior leadership and the radiation department staff. It was in the first three stages of the implementation cycle that generating interest and support from senior administrative leaders was most important in ensuring that IMRT was adopted into practice. One key informant explained that the radiation department at Centre A went to great lengths to demonstrate the benefits of IMRT first-hand to the head of the oncology program, who was a practicing surgeon, explaining that "...it was extremely helpful that senior management had an intimate understanding of the problem" (Key Informant (KI):1A). Making the implementation process a departmental objective was also expressed as important. "[R]ight from the beginning the top person of the program needs to have that ... leadership skill that says ... this isn't a choice anymore this is a must and we need to keep it moving" (KI:3C).

Similarly, strong departmental clinical leadership was identified by most of the key informants as an enabler in the first three stages of the adoption/implementation cycle. Clinical champions, who were tasked with leading the implementation initiative at the clinical department level, were usually the heads of their respective professional groups (e.g., heads of radiation oncology, physics or radiation therapy). The participation of radiation oncologists, in particular, as drivers of the implementation process was highlighted by most of the physicists and radiation therapists. As one key informant stated, "you need the rad onc buy-in up front or you're not going to get anywhere" (KI:4D). In fact, the lack of a physician champion at Centre C was identified as a contributing factor to the delay in IMRT implementation. As a Centre C key informant explained, the lack of a radiation oncology champion "is one of the reasons that the centre is behind the rest of the province. [There has not been] a keen individual who has taken this on as their main academic pursuit [because] all of us have so many fingers in so many pots" (KI:3B).

#### Collaboration

Collaboration was defined as the act of individuals or organizations working together in the interest of a common goal. Collaboration among disciplines (oncology, physics, radiation therapy), within radiation departments, and among cancer centres was consistently highlighted in the implementation phase (stage 4) by all of the key informants. Building strong relationships within the department is crucial to effective collaboration amongst the staff. At Centre A, due to the renovation of the radiation department, "physical space was changed to a more favourable layout, having it more open to allow people to collaborate more effectively" (KI:1D). This department re-design assisted in collaboration and communication between disciplines. "[B]ecause the physicists offices are next to the oncologists, we can't avoid those guys and they can't avoid us, so [there is] a constant dialogue" (KI:1E).

All four cancer centres set up implementation teams to help facilitate the process and held multidisciplinary workshops with guest speakers to introduce the IMRT implementation process and address concerns. A few of the key informants, particularly the physicists and radiation therapists, stressed the necessity of regular, interdisciplinary meetings during the implementation stage to ensure the flow of communication. For example, the full implementation team at Centre B "met on a regular basis ... just to know what the issues were, what the timelines were and to sort of develop protocols in parallel with that process, so that, when it came close to being live, everyone was well aware of how it got to that point" (KI:2D). Providing staff with regular progress updates was encouraged at three of the centres during the implementation stage in order to keep people motivated. For instance Centre A set targets and tracked the number of patients treated with IMRT, posting updates in common areas of the radiation department.

Collaboration amongst the cancer centres was also identified as an enabler by most key informants. For example, Centres A and B benefited from the presence of 'boundary spanners' (individuals with significant social ties inside and outside the centre), who formed formal and informal relationships with other centres and recruited experts nationally and internationally in order to learn from their IMRT experiences. Centre B, for instance, recognized that they did not possess sufficient physics staff and expertise to implement IMRT and decided to collaborate with another centre. A key informant from Centre B explained "[If] we tried to do it [implement IMRT] on our own then the learning curve is long, there is a risk of errors, setbacks, ...but if we go with a centre that has done this in an established way, with protocols and ... share knowledge, we could wrap this up faster" (KI:2A).

Despite collaboration efforts, a few key informants remarked that some centres continue to work in silos, as one key informant explained "...we show everything we have to everybody, it's not a secret ... but we have had more visitors from out east or from out west than from Ontario" (KI:1A). These sentiments were echoed by another key informant who encouraged collaboration with experts in the field during the implementation stage, stating that "[i]f we are going to improve health care we need to bring together appropriate individuals, we need to share knowledge. ... we can continue to do this [work independently]... but I can guarantee that we will fall behind the curve because something that should take 3 months will take 5 years" (KI:2A).

#### Training, Expertise and Standardization

Training and expertise are defined as activities that lead to a skilled behaviour, while standardization refers to the process of automating and regulating treatment procedures and protocols. Due to the steep learning curve and the potential for serious treatment error with IMRT, appropriate training and expertise were highlighted as important facilitators during the implementation stage by all key informants. Workshops, often featuring invited expert speakers, and hands-on IMRT courses were most often listed as effective enablers. The educational initiatives provided an opportunity for the radiation staff to familiarize themselves with the technology and learn about its benefits, which "demystified it a bit" (KI:1A).

Key informants from all four centres highlighted the need for adequate physics support in order to initiate the implementation process in stage 4. In particular, the radiation department at Centre B lacked experienced physicists and arranged for physics support from a more experienced centre. This arrangement was stated to be very successful, since "it was literally like bringing in an educational team ... [they] worked with the staff, built the protocols, the processes, the policies, and the procedures" (KI:2A). Centre C similarly identified the lack of IMRT experience as a barrier and one of the main reasons for the delay in implementation, however they did not seek out direct collaboration with another centre. "[W]e don't have anybody on site who has done this before so we are learning together" (KI:3B).

There was a general consensus that with time and experience IMRT becomes more efficient and less resource intensive, especially when procedures are standardized and consistently followed by the entire department. One key informant explained, "the danger with IMRT is that....everybody is doing their own thing, and nobody understands what somebody else is doing" (KI:1A). Echoing this sentiment other key informants stated that "there is no role for different physicians who want things done in a different way, it's time consuming and resource intensive" (KI:1E) and "simply not safe" (KI:1B). The introduction of standard process protocols, standard nomenclature and establishing the point at which a treatment plan is acceptable to move forward to actual treatment were suggested as solutions for reducing 'boutique' approaches and supporting IMRT implementation. "If you can get people who all believe that things have to be written down and adhered to and monitored, [there will be] much bigger chances of success" (KI:2D).

#### Resources

A resource is defined as an input, including but not limited to time, money, staff and technology, which is used to achieve an objective. The lack of certain resources, such as equipment, personnel and time were identified as potential barriers, particularly in the first three stages of the adoption/implementation cycle. The task of upgrading treatment machines and inadequate equipment were seen as barriers at all four cancer centres. Managing the clinical and implementation workloads was expressed as particularly challenging by the majority of key informants, especially due to competing programs and/or professional duties. The increased costs of implementing new technologies were also listed by a number of key informants as barriers. "[IMRT] involves more time, more staff, it involves different kinds of software and both of those ultimately translate into money" (KI:2A).

Key informants from each of the four cancer centres commented that obtaining implementation funding was a barrier. Key informants, particularly from Centre C commented that the radiation treatment funding formula, which is set by the provincial cancer agency, did not reflect the increasing complexity of current radiation treatments. Furthermore, they stated that the newly proposed complexity-based formula would create further challenges for implementing IMRT, since centres such as Centre C, who are not treating with complex technologies, will ultimately receive less funding. One key informant asked "how can you implement a new technology that is going to be more time consuming and need more resources when you are getting less [funding]?" (KI:3A). Another key informant added that "essentially it's extra work that we have to do at the end of the day" (KI:3D). Despite prevailing funding challenges, all centres were successful in securing some implementation funding through research grants, one-time funding arrangements, or special project funding.

#### Resistance to Change

Resistance to change was defined as the reluctance or unwillingness to adopt new policies, practices, or procedures. The majority of key informants agreed that resistance to change was an inevitable barrier to the adoption and implementation of new radiation technology. A number of comments suggested that radiation oncologists and older staff were more resistant to change. The most commonly cited explanation for the resistance was that some simply do not wish to change the way they practice since they are comfortable and familiar with it. "[A]round the province there are still individual radiation oncologists who like the way they always treated patients and haven't changed" (KI:3A). Key informants in all four centres also reported that there was some intimidation and/or anxiety during the initial phase of implementation of IMRT. These feelings were most evident prior to the treatment of the first patient. Overall intimidation and anxiety was most evident during the first three stages of the adoption/implementation cycle and were a result of the large learning curve, the major shift in thinking and change in practice.

However, some felt that intimidation and anxiety were not major barriers. One key informant stated that intimidation is a barrier "that I don't have a lot of experience with or sympathy for, ... [IMRT] is vastly different than the radiotherapy a lot of people trained with but ... we should be able to keep up with that change" (KI:2D). There was general agreement that the resistance phase passes as people are educated about the technology and its benefits become evident. One informant (KI:4D) added that resistance is also giving away due to pressure from other colleagues and patients, who expect high quality treatment.

#### Wait Time Policies

Wait time policies are the explicit targets set by responsible health authorities for the maximum time a patient should spend waiting for treatment (e.g., the waiting period from referral to treatment). While most key informants admitted that an individual patient's treatment will take longer with IMRT, especially in the early stages of implementation, they typically suggested that the shift to IMRT did not have a large impact on overall wait times. One key informant pointed out that "patients are very well informed ... and they are willing to take that longer wait so that they can have IMRT" (KI:2C). While it was thought that IMRT initially demanded more resources, which could impact on wait times, several key informants stated that benefits of implementing IMRT outweighed this loss. One key informant commented that wait times are "something that a lot of people throw up as a barrier but I don't think we are doing anyone a service by offering inferior treatment just to get more people through the door" (KI:2D). Some commented on the 'dichotomy of demands' between wanting to implement the latest technology and wanting to maintain wait times. "So there's this pressure between different perspectives of what constitutes quality treatment; on the one hand some people would say quality treatment is providing IMRT, others would say quality treatment is providing treatment in ... the shortest possible time and so we have to balance those demands" (KI:4A).

#### Scientific Evidence

In this study scientific evidence refers to methodologically rigorous research, such as randomized controlled trials (RCTs). Key informants were asked to comment on the type of evidence used during stage 1 of the adoption/implementation cycle and on the challenges associated with the evaluation of radiation technology (stage 5), particularly using randomized controlled trials. Although evidence, obtained from RCTs, supporting the use of IMRT was not available, this, for the most part, was not viewed as a barrier and did not hinder the implementation of IMRT. Rather, indirect evidence, or what some called 'technical evidence' derived from IMRT's ability to deliver higher doses to the tumour while sparing healthy tissues and evidence of reduced toxicity and improved quality of life was used to support the adoption and implementation decisions. There were, however, some differences in how the participants discussed the role of evidence, where the physicists and radiation therapists were more likely to highlight the varied advances in technology or the 'technical evidence', the radiation oncologists were quick to point out issues of equipoise with RCTs and focused on the various toxicity and quality of life studies as evidence of IMRT benefit.

## Discussion

**Figure 3:**
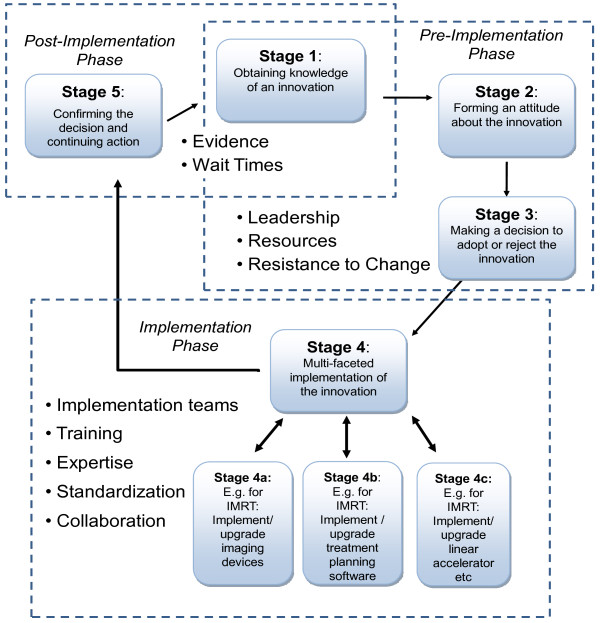
**A framework for multifaceted adoption/implementation of complex and evolving technologies (Adapted from Rogers 1995)**.

The adoption and implementation of complex and evolving technologies is a multifaceted process that requires a multi-disciplinary implementation team, a clearly defined implementation strategy and a comprehensive evaluation phase. Since the modern radiation department is constantly reinventing and evolving treatment approaches, rather than simply replacing them, these departments are in an ongoing state of technology implementation. It is in fact a new treatment 'program' that is being implemented, rather than simply the utilization of a new piece of equipment. To guide decision-makers, the adaptations made to Roger's innovation adoption model at the outset of the study (Figure 1) were further modified to reflect the impact of the identified enablers and barriers on the decision to adopt and implement technological innovations, such as IMRT (Figure 3). In the refined model, the key factors identified in this study have been linked to the stages of implementation where they have the greatest impact.

Stages 1 to 3 of the adoption/implementation cycle are primarily concerned with the adoption decision and broadly categorized as the pre-implementation stages. The gathered data suggest that factors which will be most influential at stages 1 to 3 include leadership, available resources, funding and resistance to change. At stage 4, when the decision to implement has been accepted, it is often only then that the technology's complexity is fully acknowledged and the multiple aspects of the implementation are specified. The post-implementation stage addresses the evolving use of the technology, demonstrating the need for continuous evaluation in light of emerging scientific evidence. While not viewed as barriers, wait times policies and the lack of scientific evidence may impact external policies, therefore, these factors have been incorporated into the framework, overlapping both stage 1 and 5 where they have the greatest potential to impact the decision to adopt or upgrade technology.

The results of this study build on and reinforce many of the implementation barriers and facilitators identified in the literature [15]. However, this study also sheds light on the high degree of complexity and variability that radiation oncology programs regularly face when implementing technologies, such as IMRT. For instance, appropriate leadership and champions have been widely documented as implementation facilitators in the literature [15,28-30] however in the radiation treatment context this task seems to be complicated by the presence of a multi-professional team that is responsible for separate yet integrated tasks. In the four cancer centres studied, the physicists, who typically were most aware of the developments in the radiation field, initiated the adoption of a new technology and were consequently identified as implementation leads. However, their efforts were often stalled if radiation oncologists, the other opinion leaders, were not on board. This type of dual leadership dynamic is not often present in the implementation of drugs or surgical innovations and poses unique challenges for radiation programs, which require further investigation. Ferlie et al, have similarly called for additional research on the boundaries among professional groups, individual professionals and associated communities of practice and their impact on the spread of innovations [22].

The resistance to change from the medical leadership may stem from the fact that the two professions seem to view evidence differently. The physicists considered IMRT as a natural progression of an evolving technology while the radiation oncologists were seeking evidence of benefit in the form of high level RCTs. Interestingly a radiation oncologist leader at Centre C was the only key informant who indicated that the evidence may not be strong enough to move forward with IMRT, which created a leadership vacuum that contributed to a long delay in implementation. Considering that RCTs are difficult and often unlikely to be carried out for most radiation treatment modalities [31], a discussion of what constitutes evidence for complex and evolving technologies, such as IMRT, is necessary to engage physicians at an earlier stage and avoid implementation delays.

The training and expertise factors also differ around evolving and complex technologies, not only because they require the acquisition of new skills by an entire team but also because these skills are not easily acquired through didactic training. Williamson et al report that due to the rapid technological developments in radiation the available quality assurance guidance for physicists is often incomplete or out of date [32]. This study demonstrated that collaboration between centres, where hands-on-training was provided by a team of experts, was an effective mode of knowledge exchange, particularly for the physicists. This type of educational outreach has not been covered in the literature however; it seems to be an initiative that a centralized cancer system may want to embrace as an effective jurisdiction wide educational strategy.

Unlike the administration of drugs which are streamlined and to an extent predictable, complex and evolving technologies, such as IMRT or surgical procedures, have the potential to create 'boutique treatments' where each physician treats patients in accordance with his or her own personal preferences. This type of treatment was identified as not only time consuming but potentially dangerous by the key informants, who stressed the standardization of treatment protocols and the introduction of a standard nomenclature within a department, as well as at the jurisdictional level.

While this study focused only on cancer centres located in Ontario, the case selection approach did ensure that the four cases varied on key criteria that allow the findings to be compared across fairly diverse settings and facilitate generalization to other contexts. For instance, the findings in this study may be of relevance to the cancer system in the United Kingdom, which is facing similar challenges to those seen in Ontario in 2007. In a recent publication Williams et al stated that "radiotherapy services in England are at different stages of developing and delivering IMRT" adding that while some centres have fairly comprehensive IMRT portfolios others have yet to begin treating patients [33]. The findings in this study will undoubtedly be of assistance in overcoming some of the implementation obstacles these departments are facing.

While radiation departments in the United States have been delivering IMRT treatment for over a decade, the issue of adoption of expensive and marginally proven technologies has recently become a common topic of discussion. Increasingly comparative effectiveness research has been promoted as an alternative means of providing an acceptable evidence base for policymaking decisions. Wallner et al suggests that the comparison of devices and procedures that are both costly and have a high clinical impact, such as proton-beam therapy and IMRT for early-stage, low-risk to intermediate-risk prostate cancer, may be beneficial [34].

Overall, these findings provide a comparative base for the implementation of other health technology innovations, such as robotic surgery, pharmacogenomic technologies and electronic medical records (EMR). For example, Finan et al state that the majority of research around robotic surgery has focused on clinical outcomes rather than factors associated with technical problems related to the implementation of new technology and equipment [35]. Similarly, within the relatively new field of personalized medicine, Freedman et al have called for "[d]issemination studies that focus on barriers and facilitators to wide-scale adoption of proven pharmacogenomic technologies or on the overuse or misuse of technologies that have questionable risk to benefit profiles" [36]. Finally, the implementation of EMRs has been described as "varied and sometimes negative, notably in public health care systems, and where the EMR is part of a larger health information system" [37]. Despite differences in underlying technical characteristics, the issues faced by many complex and evolving technologies are similar to IMRT, therefore some of the lessons learned in this study are potentially transferable and may provide further guidance and insights on how to more effectively and efficiently make challenging adoption/implementation decisions.

## Conclusions

This study focused on an in-depth examination of the adoption and implementation of IMRT in publicly funded cancer centres. A key point underlying this work is that complex and evolving technologies are never really one type of technology, but rather reflect multiple inter-related techniques and processes. Hence, the framework presented in this study may assist in identifying barriers and facilitators in the implementation of different types of innovative radiation therapies, for instance brachytherapy or stereotactic radiotherapy, as well as other technologies for which there is a paucity of implementation research. The factors identified in this study may assist in the improvement of workflow, safety, efficiency, and equitable access to appropriate care.

## Competing interests

The authors declare that they have no competing interests.

## Authors' contributions

KB contributed to the conception and design of the study, collected the data, led the data analysis and interpretation, and prepared the initial draft of the manuscript. MJD has made substantial contributions to the conception and design of the study, interpretation of data and in revising the manuscript critically for important intellectual content. DH and AW have been involved in revising the manuscript critically for important intellectual content. All authors read and approved the final manuscript.

## Pre-publication history

The pre-publication history for this paper can be accessed here:

http://www.biomedcentral.com/1472-6963/11/178/prepub
